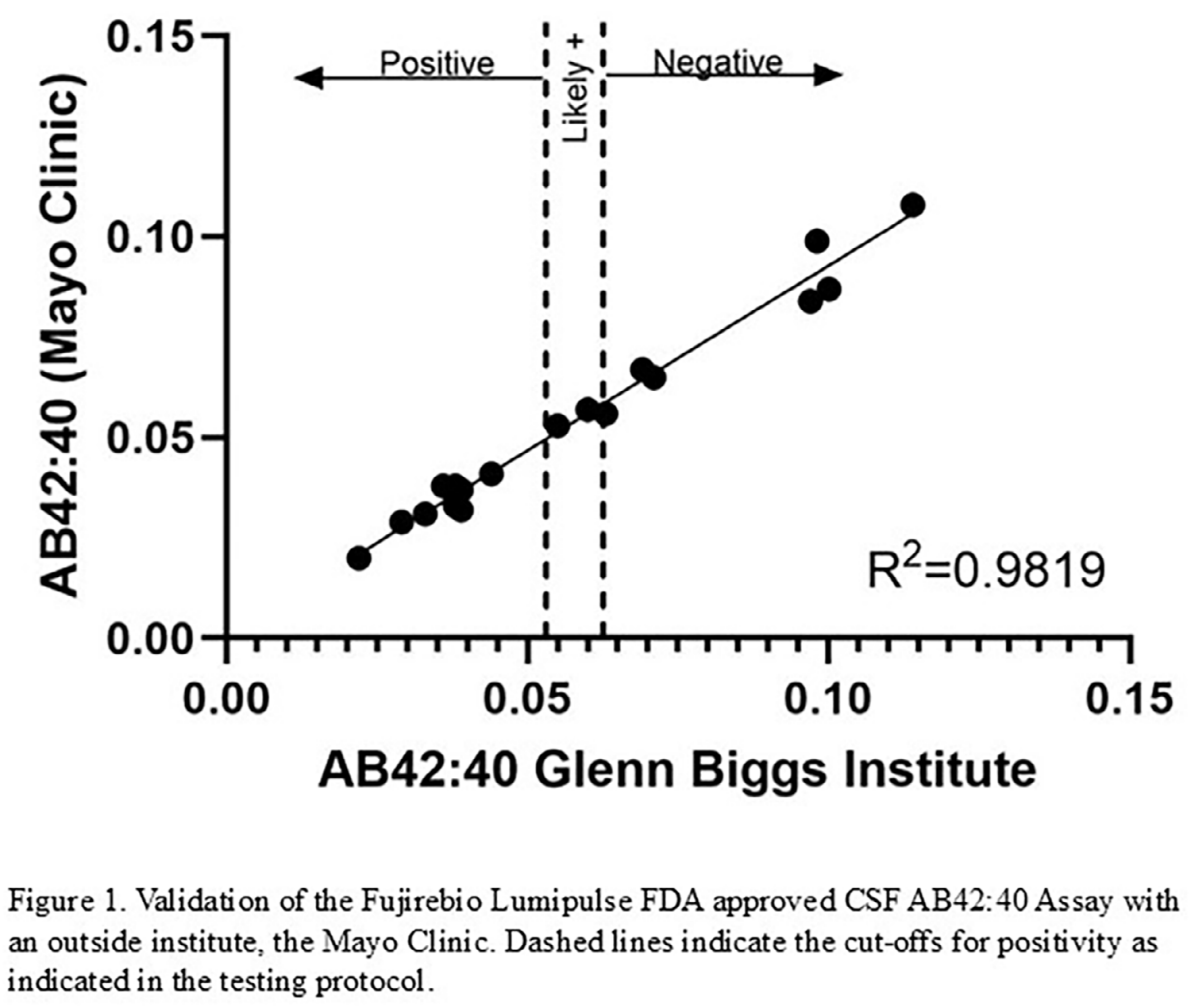# CLIA Certification for Alzheimer's Biomarkers at the Glenn Biggs Institute in San Antonio, TX

**DOI:** 10.1002/alz70856_101042

**Published:** 2025-12-25

**Authors:** Julie Parker‐Garza, Tiffany F. Kautz, Sudha Seshadri, Eric L. Shipp, Oluwaseun Ogunbona, Margaret E Flanagan

**Affiliations:** ^1^ Glenn Biggs Institute for Alzheimer's & Neurodegenerative Diseases, UT Health San Antonio, San Antonio, TX, USA; ^2^ Glenn Biggs Institute for Alzheimer's and Neurodegenerative Diseases, University of Texas Health Science Center, San Antonio, TX, USA; ^3^ Glenn Biggs Institute for Alzheimer's & Neurodegenerative Diseases, University of Texas Health Science Center, San Antonio, TX, USA; ^4^ Glenn Biggs Institute for Alzheimer's & Neurodegenerative Diseases, University of Texas Health Science Center, San Antonio, TX, USA

## Abstract

**Background:**

The Clinical Laboratory Improvement Amendments (CLIA) regulate laboratory testing to ensure accuracy, reliability, safety, and timeliness of results. Biomarkers are becoming crucial for early detection, diagnosis, monitoring, and personalized treatment of Alzheimer's disease (AD). CLIA approval is necessary for Alzheimer's biomarkers to be returned to patients and integrated into routine clinical practice. Here, we explain the process and requirements for CLIA AD biomarker approval for the newly FDA approved Fujirebio Lumipulse amyloid‐beta (AB) 42:40 cerebral spinal fluid (CSF) assay.

**Method:**

We reviewed the CLIA approval process, focusing on its application to AD biomarkers. We performed required validation protocols for the AB 42:40 ratio for CSF on the Lumipulse G1200 analyzer and ensured that we met or exceeded the staffing, monitoring, and documentation requirements for a Certificate of Compliance CLIA certification. CSF from the biobank program at the Glenn Biggs Institute was used to assess the reliability of this test in two laboratory settings, UT Health San Antonio Glenn Biggs Institute and Mayo clinic, by blinded laboratory technicians.

**Result:**

CLIA approval for the FDA approved Fujirebio AD biomarker test was contingent upon demonstrating test accuracy, reproducibility, and clinical utility. We were able to achieve CLIA certification after meeting each of these requirements. Our validation testing showed high correlation (R2=0.9819) between the two laboratory sites (Figure 1).

**Conclusion:**

With our certificate of compliance, we can now return Fujirebio Lumipulse AB 42:40 biomarker results to clinicians and research studies to aid in clinical diagnosis of AD. CLIA approval guarantees that biomarker assays demonstrate consistent performance in clinical settings, addressing variability across different laboratories and patient populations, which we found to be true for the Fujirebio CSF AB42:40 biomarker test. CLIA approval is an essential step in making AD biomarkers accessible for routine clinical application, as well as precision medicine approaches and enhancing early diagnosis and intervention strategies for Alzheimer's Disease.